# RAGE Expression in Human T Cells: A Link between Environmental Factors and Adaptive Immune Responses

**DOI:** 10.1371/journal.pone.0034698

**Published:** 2012-04-11

**Authors:** Eitan M. Akirav, Paula Preston-Hurlburt, Justin Garyu, Octavian Henegariu, Raphael Clynes, Ann Marie Schmidt, Kevan C. Herold

**Affiliations:** 1 Departments of Immunobiology and Internal Medicine, Yale University, New Haven, Connecticut, United States of America; 2 Department of Medicine, Columbia University, New York, New York, United States of America; 3 Department of Medicine, New York University, New York, New York, United States of America; La Jolla Institute for Allergy and Immunology, United States of America

## Abstract

The Receptor for Advanced Glycation Endproducts (RAGE) is a scavenger ligand that binds glycated endproducts as well as molecules released during cell death such as S100b and HMGB1. RAGE is expressed on antigen presenting cells where it may participate in activation of innate immune responses but its role in adaptive human immune responses has not been described. We have found that RAGE is expressed intracellularly in human T cells following TCR activation but constitutively on T cells from patients with diabetes. The levels of RAGE on T cells from patients with diabetes are not related to the level of glucose control. It co-localizes to the endosomes. Its expression increases in activated T cells from healthy control subjects but bystander cells also express RAGE after stimulation of the antigen specific T cells. RAGE ligands enhance RAGE expression. In patients with T1D, the level of RAGE expression decreases with T cell activation. RAGE+ T cells express higher levels of IL-17A, CD107a, and IL-5 than RAGE− cells from the same individual with T1D. Our studies have identified the expression of RAGE on adaptive immune cells and a role for this receptor and its ligands in modulating human immune responses.

## Introduction

Adaptive T cell responses are modified by T cell activation signals delivered through the T cell receptor (TCR) and costimulatory ligands, as well as environmental factors [1], [2]. The effects of cytokines on T cell differentiation have been appreciated for many years, but nutrients including glucose, metabolites, and other molecules such as products of cell death may affect the activation signals and transcriptional machinery that control cell differentiation [3], [4]. These factors, which serve as *modulators* rather than primary initiators of immune responses, have not been well studied; in part because their role may be defined by the setting of the immune responses *in vivo* which is difficult to recreate *in vitro*. For example, cytokines that are products of an activated immune response may lead to activation induced cell death of T cells, but nutrient deprivation may be an equally important factor leading to the death of T cells in tumors or ischemic tissue [5], [6]. In settings of autoimmunity, these environmental factors may be particularly important since destruction of organ tissues such as thyroid, adrenal, or the islets of Langerhans may change the environment. The importance of hyperglycemia, following destruction of β cells may be reflected by the more rapid decline in β cell function in T1D after the onset of hyperglycemia compared to prior to hyperglycemia, and the amelioration of β cell decline with tight glycemic control in the Diabetes Control and Complications Trial [7].

One of the molecules that may play a pivotal role in linking environmental factors and immune responses is the Receptor for Advanced Glycation Endproducts (RAGE) [8], [9], [10], [11]. RAGE was originally identified as a receptor for glycosylated proteins and has been postulated to be involved in the pathogenesis of secondary end organ complications of diabetes [12], [13], [14], [15], [16], [17]. It is expressed on parenchymal tissues including pulmonary alveoli and endothelial cells where it is thought to participate in atherogenesis [18], [19], [20], [21], [22], [23], [24], [25], [26], [27], [28], [29]. In addition to glycated proteins, RAGE binds molecules such as HMGB1, S100b, calgranulins and others [30], [31], hence its designation as a scavenger receptor that may play a role in immune responses at sites of tissue destruction. Its ability to bind ligands found at sites of cell death and inflammation (so called “damage associated molecular patterns” or DAMPs) has led many to conclude that RAGE is involved in immune responses associated with these events, and may modulate inflammatory and adaptive immune responses [31]. RAGE activation has been shown to play a role in diverse settings including sepsis and atherosclerosis as well as disease processes including diabetic nephropathy, rheumatoid arthritis, and Alzheimer's disease and hypoxia/reoxygenase injury [11], [18], [32], [33], [34], [35], [36], [37], [38], [39], [40], [41], [42], [43].

RAGE is a type I transmembrane protein composed of three extracellular immunoglobulin-like domains (V, C1, and C2), a single transmembrane domain and a short cytoplasmic tail thought to be important in signal transduction [19], [31], [44], [45]. Signaling through RAGE induces several intermediaries including NF-κB, MAPKs, PI3K/Akt, Rho GTPases, Jak/STAT, and Src family kinases [8], [21], [46], [47], [48], [49], [50] [45]. RAGE is found on human and murine antigen presenting cells even in the absence of inflammation [27], [51], [52], [53], [54]. Some investigators have described a cooperative relationship between RAGE and TLR 2, 4, and 9 activation as well with the IL-1 receptor [54], [55], [56], [57]. [57].

In murine models, we identified RAGE on activated T cells and were able to modulate diabetogenic T cell responses with sRAGE. A small molecule inhibitor of RAGE, TTP488, delayed islet allograft rejection in BALB/c mice and RAGE−/− mice showed delayed rejection of islet allografts consistent with more recent studies showing that HMGB1 and RAGE are involved in islet graft loss [58], [59], [60]. These studies identified a role of RAGE on the differentiation and activation of murine T cells [61].

There is, however, no information about RAGE expression and function on human T cells despite a predicted effect and support from preclinical studies. We therefore, studied expression and function of RAGE on human T cells in patients with diabetes in whom RAGE ligands are increased, and in healthy control subjects. We found that RAGE is constitutively expressed in T cells from patients with diabetes. RAGE+ T cells have a skewed phenotype suggesting that environmental RAGE ligands may affect adaptive immune responses.

## Results

### Intracellular RAGE expression is increased in activated T cells from healthy control subjects

**Figure 1:**
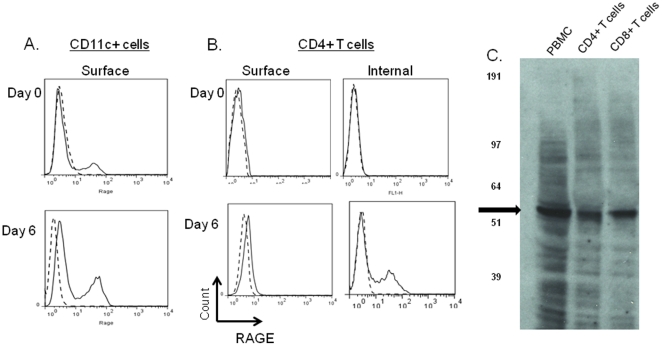
Expression of RAGE on human APC's and T cells. A: Surface RAGE expression was studied on CD11c+ PBMC before (top) and after (bottom) culture with LPS for 7 days. (solid line = staining with anti-RAGE antibody, dashed line = staining with isotype control) B: Cell surface (L and intracellular RAGE expression was studied on CD4+ T cells before (top) and after 7 days in culture with anti-CD3 mAb (bottom). A single experiment representative of cultures with more than 4 donors is shown. C: PBMC were activated with anti-CD3 mAb for 48 hrs and lysed or separated into CD4+ and CD8+ T cells with magnetic beads and lysed. A blot of the lysates was probed with anti-RAGE antibody. The arrow identifies RAGE in the cells.

We first studied the expression of RAGE on human T and CD11c+ cells in peripheral blood from healthy control subjects under basal conditions and after activation. Consistent with previous reports, we identified RAGE surface expression on CD11c+ cells, which increased following activation of the cells for 6 days with LPS (Figure 1A). We did not detect RAGE expression on resting T cells. Moreover, activation of resting T cells with anti-CD3 mAb, also failed to increased RAGE expression on the surfaces of the T cells. However, intracellluar staining of T cell revealed RAGE expression in activated cells (Figure 1B). RAGE was expressed in both CD4+ and CD8+ T cells at similar levels. We confirmed the expression of RAGE on activated CD4+ and CD8+ T cells by staining on Western blot (Figure 1C).

**Figure 2:**
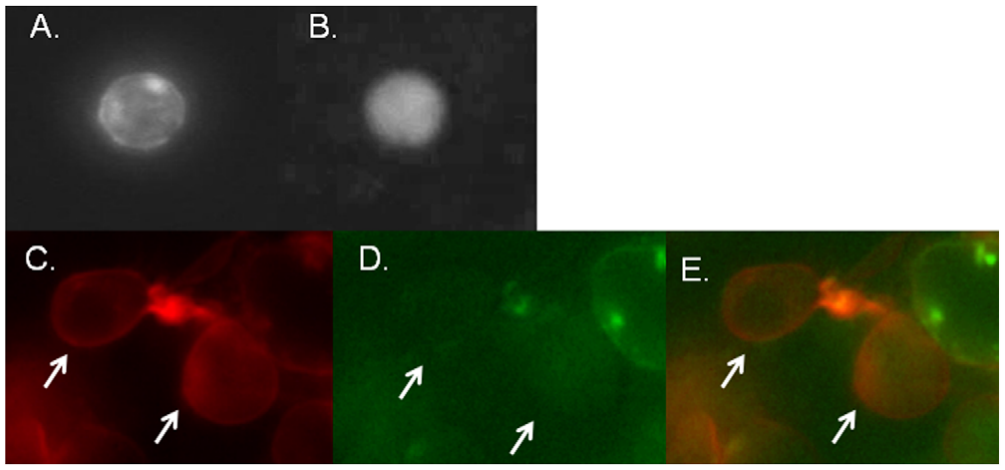
RAGE is seen in a granular pattern in T cells and colocalizes with endosomes. A: Jurkat cells were transfected with GFP-RAGE (A) or control GFP vector (B) and photographed. A granular pattern of staining is seen within the RAGE transfected cells. C–E: HEK293 cells were transfected with GFP-RAGE (D and E) and fixed and stained with RhoB (C and E). Panel E shows the merged staining. The arrows indicate cells+ for RhoB and RAGE.

To determine the intracellular location of RAGE expression, we transfected HEK293 and Jurkat cells with GFP-RAGE. In living Jurkat cells, RAGE staining appeared as a granular pattern in the cytoplasm. We co-stained transfected HEK 293 cells (with RHOB-N-RFP) and found co-localization of RAGE with early endosomes (Figure 2).

### T cell activation and RAGE ligands enhance RAGE expression on T cells

**Figure 3:**
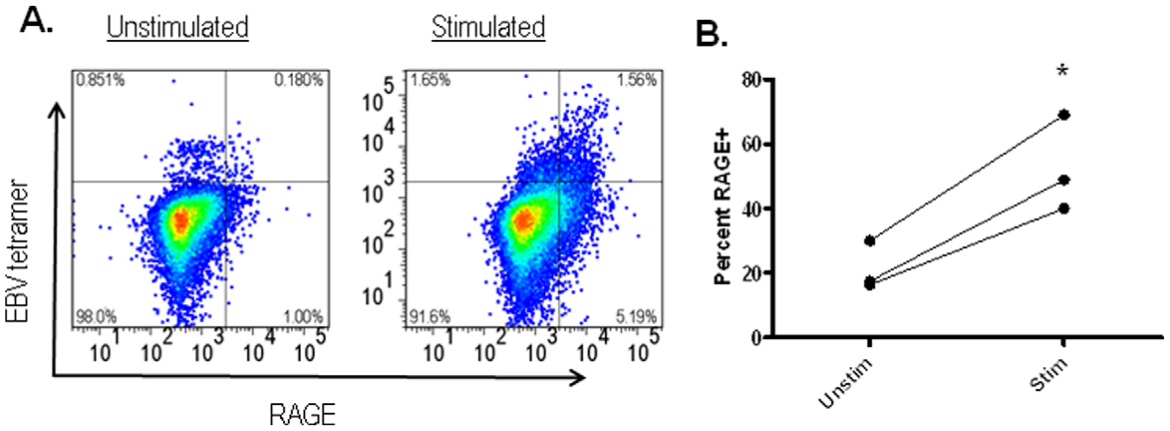
RAGE is expressed on antigen specific CD8+ T cells during culture. Peripheral blood cells from HLA-A2.1+ healthy control subjects that were cultured with or without EBV peptide were stained with Class I MHC tetramer loaded with EBV peptide and for intracellular RAGE. Culture with the peptide increased the proportion of tetramer+ T cells increased 2.5-fold. On the tetramer+ T cells, the proportion that were RAGE+ increased 2.5 fold (p = 0.02). Data from 3 individuals are shown.

Our findings with anti-CD3 mAb suggested that antigen stimulation induces RAGE expression on T cells. To test this directly, we cultured PBMC from HLA-A2+ subjects with or without EBV peptide for 7 days. The antigen specific T cells were identified by staining with Class I MHC (HLA-A2.1) tetramers in the two cultures. Compared to the cultures without peptide, the cultures with peptide showed an increase of 2.5±0.15 fold in the percentage of EBV-reactive CD8+ T cells. In addition, a greater proportion of tetramer+ cells were also RAGE+ (21.3±4.36% vs 52.6±8.58%, p = 0.02) (Figure 3).

**Figure 4:**
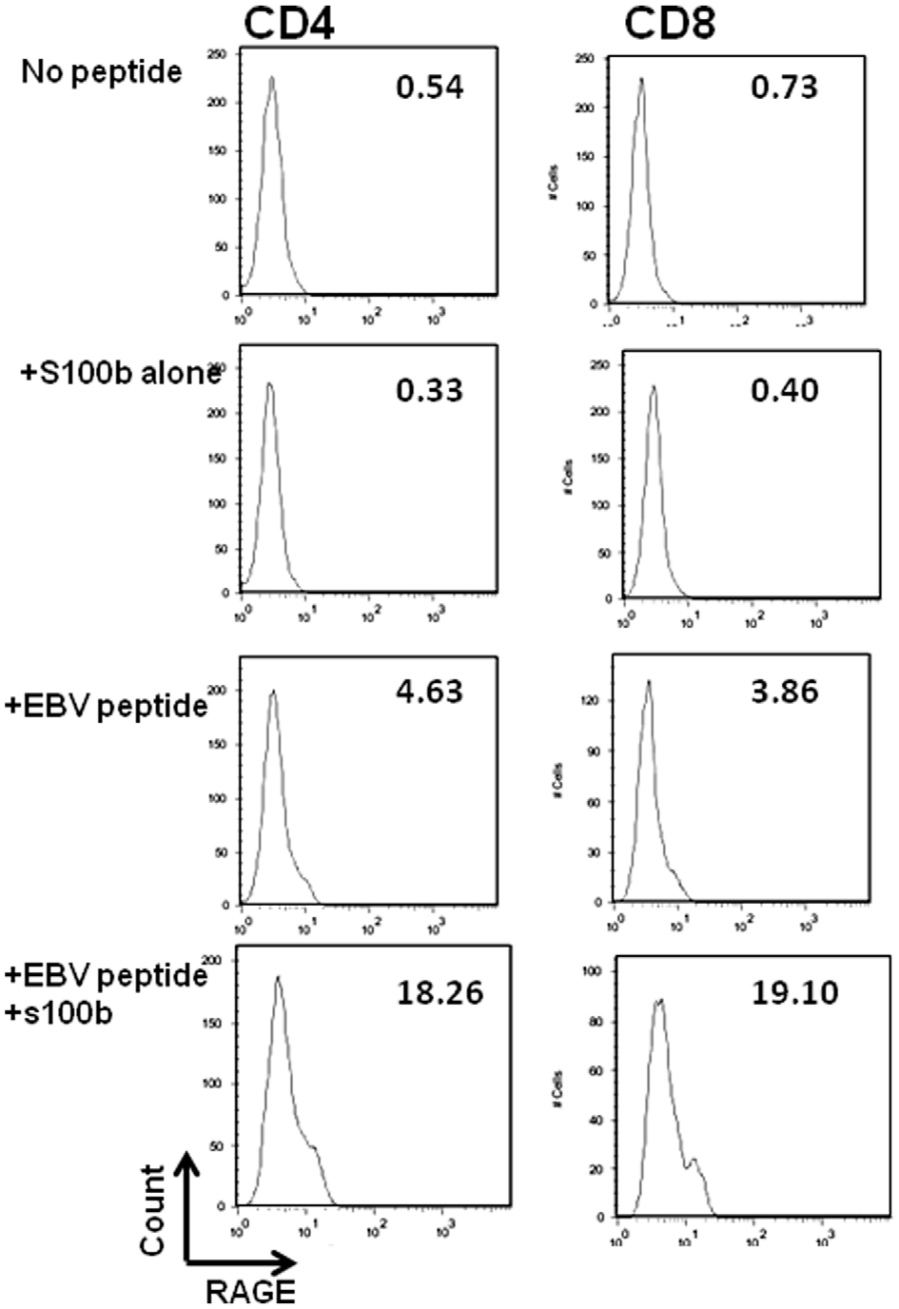
RAGE ligand enhances RAGE expression on T cells. Peripheral blood cells were cultured with or without EBV peptide and IL-2 with or without S100b. After 7 days, CD4+ and CD8+ T cells were analyzed for the expression of RAGE. The percentages shown in each panel indicate the percentage of RAGE+ T cells (minus background staining with control Ig) of CD4+ or CD8+ cells. A single experiment representative of 3 is shown.

Interestingly, we also found increased RAGE expression on other CD8+ T cells in the cultures suggesting that factors other than direct TCR stimulation were responsible for RAGE expression. The most likely factor(s) were RAGE ligands themselves, and therefore to examine this in more detail, we tested the effects of RAGE ligands and TCR activation on the expression of RAGE on other T cells during the cultures. We cultured PBMC from a (HLA-A2.1+) healthy control subjects with HLA-A2.1-restricted EBV peptide in the presence or absence of the RAGE ligand S100b, and studied the expression of RAGE in CD4+ as well as CD8+ T cells (Figure 4). Consistent with our findings in Figure 3 in the antigen specific T cells, there was an increase in the number of cells that expressed RAGE after 7 days in culture with peptide on unselected CD8+ T cells but also on “bystander” CD4+ T cells. When S100b was added to the cultures, the proportion of cells expressing RAGE was increased more than 25 fold whereas culture with S100b alone did not increase the proportion of RAGE+ T cells.

### T cells from patients with T1DM express intracellular RAGE under basal conditions

**Figure 5:**
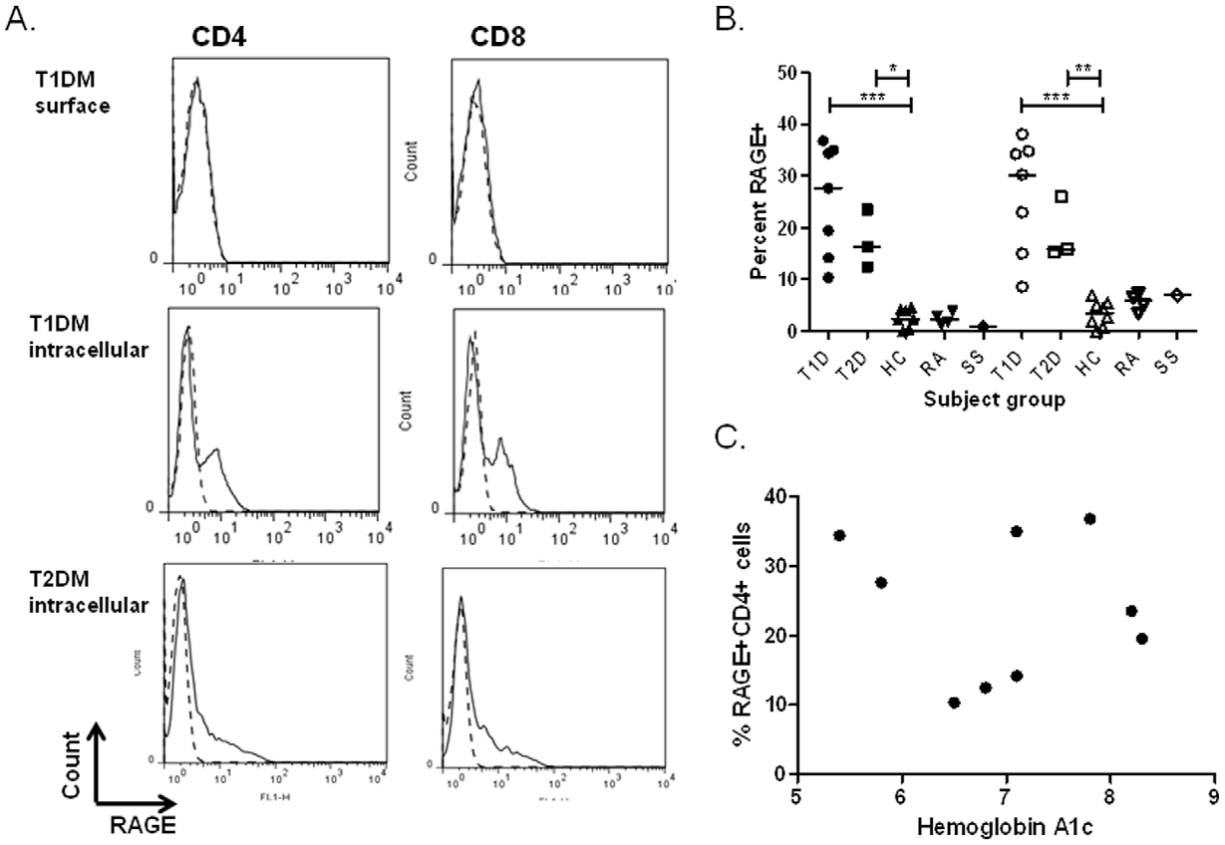
Expression of RAGE on T cells from patients with T1D and T2D. A:PBMC were isolated from patients with T1D (top two rows) and T2D (bottom row). They were stained with CD4+ or CD8+ Abs and for surface or intracellular RAGE. Two single experiments, representative of 7 are shown. B. The level of RAGE expression in unmanipulated CD4+ (solid symbols) and CD8+ (open symbols) T cells from patients with T1D, T2D, and healthy control subjects (HC) or patients with rheumatoid arthritis (RA) and Sjogren's syndrome (SS) are shown (*p<0.05, *** p<0.01). C. The relationship between hemoglobin A1c levels and the percentage of RAGE in CD4+ T cells in patients with T1 and T2D is shown (p = ns).

These findings indicated that RAGE ligands enhanced RAGE expression on activated T cells. We reasoned that since RAGE ligands are found in the serum of patients with diabetes, RAGE expression might distinguish T cells from patients, and therefore examined RAGE expression on peripheral blood T cells from patients with Type 1 (T1D) and Type 2 diabetes (T2D). Similar to our findings in healthy control subjects, we did not detect RAGE expression on unstimulated cells from patients. However, we found that the unstimulated cells from diabetic patients with T1D and T2D diabetes expressed intracellular RAGE on CD4+ and CD8+ T cells (Figures 5A and B). The level of expression in freshly isolated cells was similar to that found on T cells from healthy control subjects that had been activated with anti-CD3 mAb (p<0.001 vs staining of unstimulated cells from healthy control subjects), and was not directly related to the ambient glucose level reflected by the hemoglobin A1c (Figures 5B and C) or duration of disease (not shown). RAGE expression was not a general feature of autoimmunity or inflammatory conditions because it was not found on unstimulated T cells from patients with rheumatoid arthritis or Sjogren's syndrome (Figure 5B).

**Figure 6:**
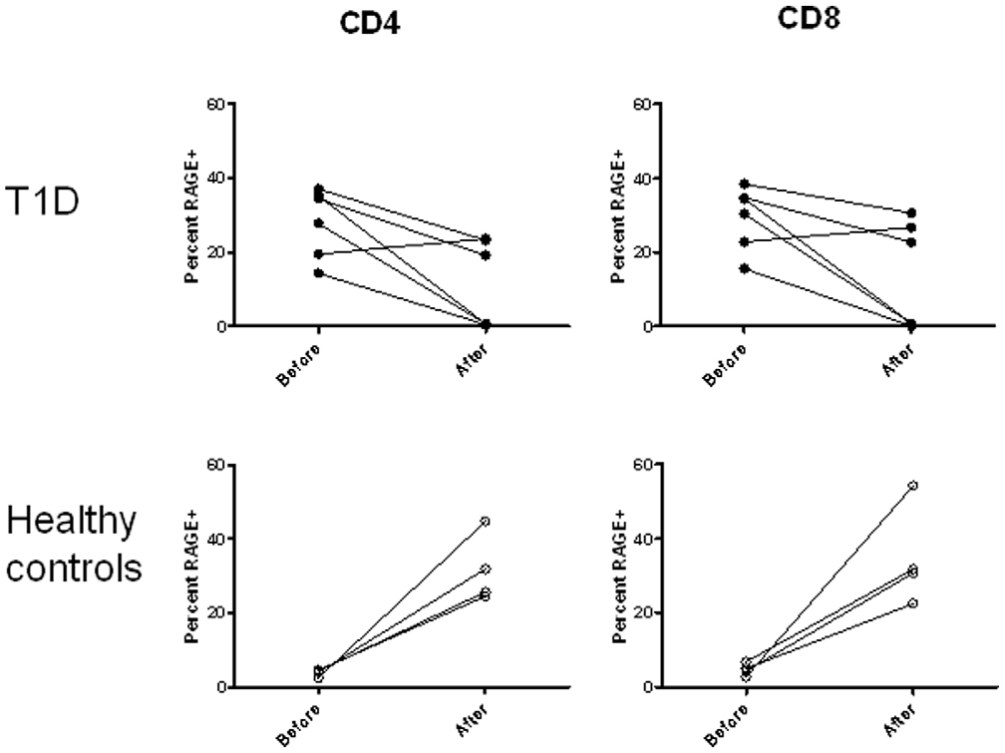
Changes in RAGE expression on activated T cells from patients with T1D and healthy control subjects. RAGE expression was studied on CD4+ or CD8+ T cells before and 48 hrs after culture with anti-CD3 mAb. RAGE expression was higher on CD4+ (p<0.001) and CD8+ (p<0.001) T cells from patients with T1D vs healthy controls. While the level of RAGE expression increased in CD4+ and CD8+ T cells from healthy control subjects (p<0.05), it decreased in the patients with T1D (p<0.05).

In addition to the basal levels, the effects of T cell activation on RAGE expression also differed in healthy control subjects and patients. In healthy control subjects the level of RAGE expression increased from 3.8±0.5% to 31.7±4.6% of CD4+ (p = 0.01) and from 4.7±0.85% to 34.8±6.79% (p = 0.03) on CD8+ T cells following culture with anti-CD3 Ab, whereas it decreased from 28.0±3.8% to 11.3±4.8% (p<0.05) on CD4+ and 29.4±3.5% and 13.7±5.9% on CD8+ T cells in patients with T1D (p<0.05) (Figure 6).

### RAGE+ T cells have a naïve phenotype but produce increased levels of IL-17

**Figure 7:**
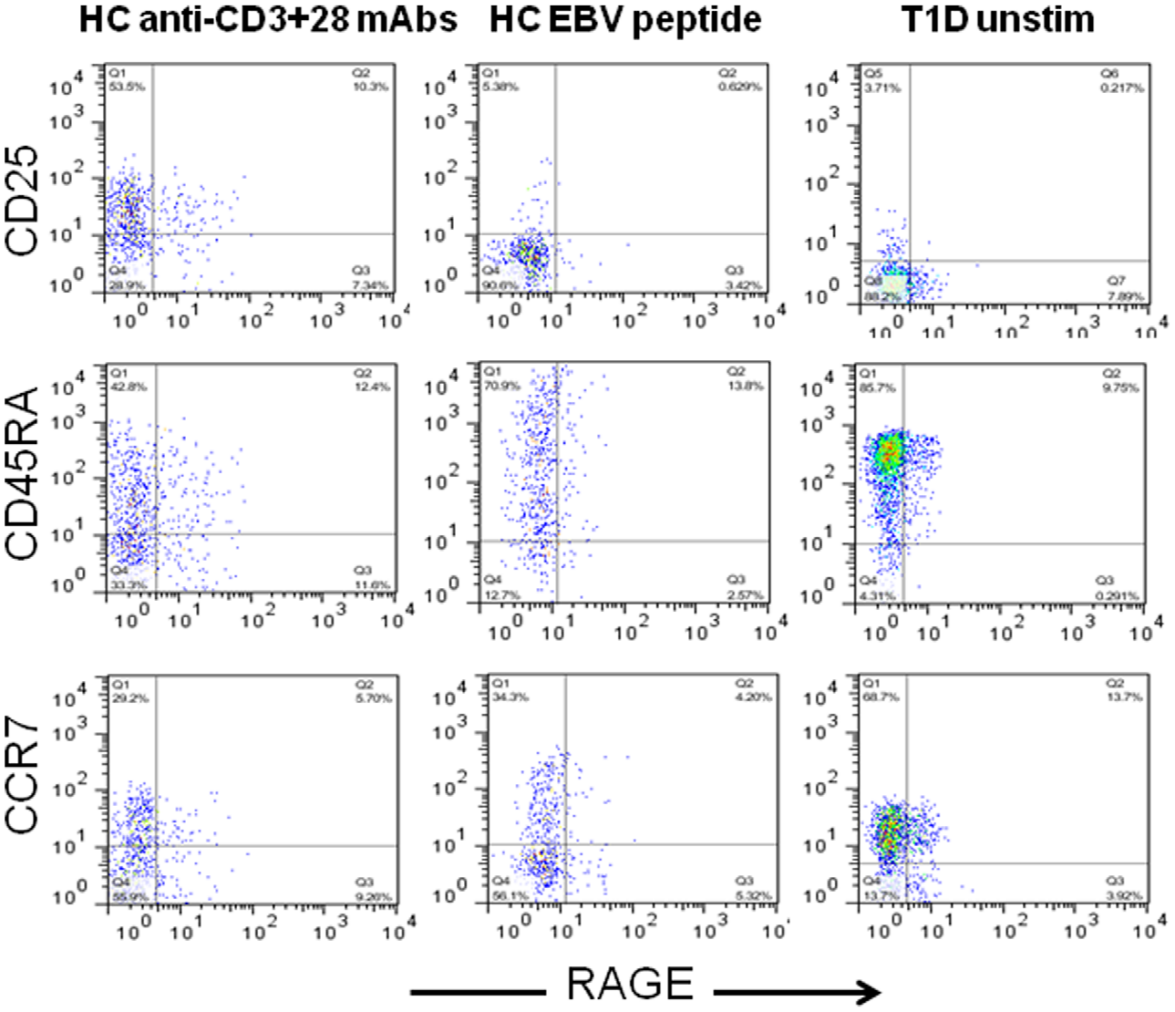
Phenotype of RAGE+ T cells. CD8+ T cells, that were not activated from a patient with T1D (R column) or a CD8+ T cells from a HLA-A2+ healthy control subject, activated with anti-CD3+28 mAbs (L column) or from the same HC subject activated with EBV peptide (middle column) were compared. The RAGE+ T cells from the patient with T1D do not express CD25, are CCR7+ and have a more uniform distribution of CD45RA. Results from a single donor representative of 3 is shown.

To understand the significance of RAGE expression on T cellular function, we studied the phenotype of RAGE+ and RAGE− CD4+ and CD8+ T cells from patients with diabetes. The majority of the RAGE+ cells were CD45RA+ and CCR7+ similar to RAGE− cells. The CD8+ T cells were CD25− whereas 30–45% of the RAGE+ CD4+ T cells were CD25+ suggesting prior activation (Figure 7).

**Figure 8:**
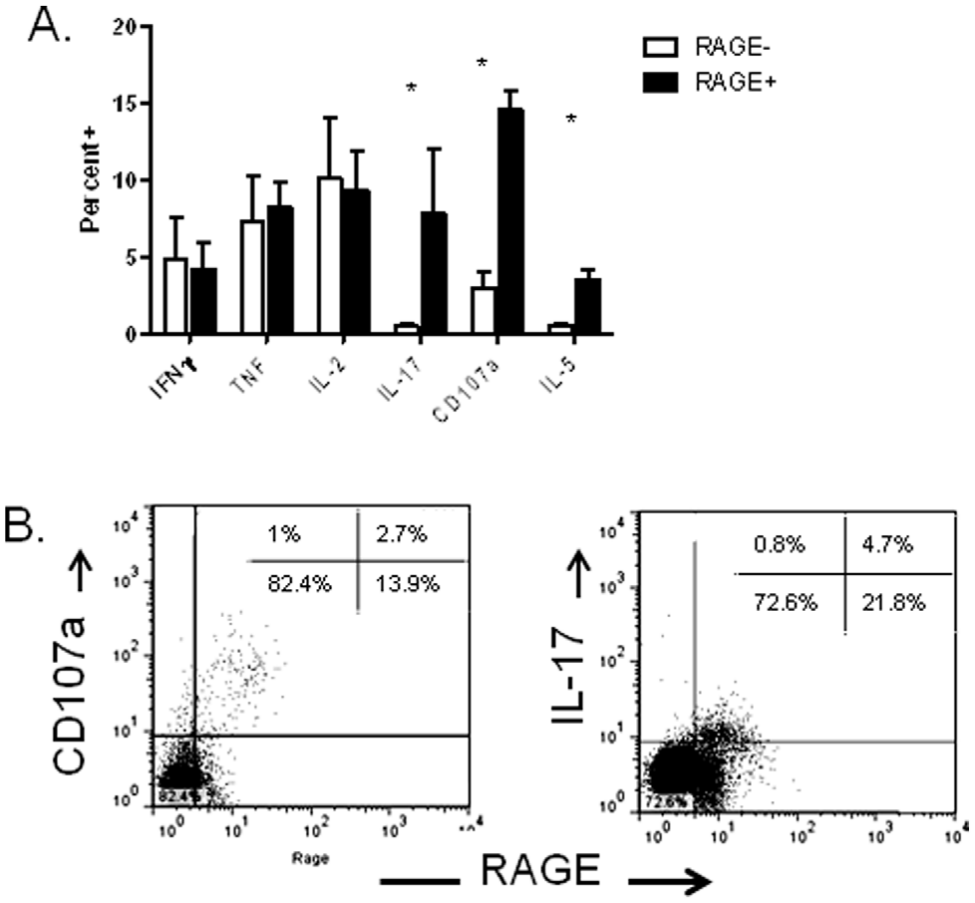
Phenotype of RAGE+ T cells. A: The phenotype of RAGE+ and − PBMC from patients with T1D were studied by flow cytometry (n = 4). PBMC were activated with PMA/ionomycin for 6 hours and the percentage of cytokine+ RAGE+ or RAGE− T cells was determined in the same individual and compared by paired t-test. The percentages that are shown (mean± SEM) represent the percent of the RAGE+ or RAGE− T cells that were cytokine+. (*p<0.05). B. A single representative experiment showing staining with RAGE and CD107a and IL-17 are shown. Gates were placed around CD4+ T cells. The inserts show the the percentage of total CD4+ cells in each quadrant.

We then studied the secretion of cytokines by RAGE+ and − T cells. We activated PBMC from patients with T1D with PMA/ionomycin for 6 hrs and compared the percentage of cytokine+ cells in the RAGE+ and − CD4+ and CD8+ T cell subsets (Figures 8A and B). There was a greater proportion of IL-17+, CD107a, as well as IL-5+ CD4+ T cells in the RAGE+ vs RAGE− subsets (Figure 8A). A similar trend was seen for IL-17, CD107a, and IL-5 among CD8+ T cells (not shown).

## Discussion

We have found RAGE expression in human T cells after activation in healthy control subjects and under resting conditions in patients with diabetes. Unlike antigen presenting cells, in which RAGE is expressed on the cell surface and in granules, RAGE expression in T cells was intracellular and colocalized with endosomes. Increased RAGE expression in diabetes is most likely due to the availability of RAGE ligands in these patients since it was found on T cells in patients with T1D and T2D but not in T cells from patients with other autoimmune diseases, and RAGE expression was further enhanced in activated T cells in the presence of RAGE ligands. The RAGE+ T cells generally displayed a naïve phenotype but a percentage of CD4+ T cells showed increased expression of CD25 suggesting prior activation and consistent with the need for TCR activation to induce RAGE on T cells. Finally, RAGE expression also coincided with higher levels of IL-17, IL-5, and CD107a compared to RAGE− T cells. These findings suggest a new mechanism whereby RAGE ligands, commonly found at sites of inflammation and in diabetes, may modulate adaptive immune responses.

Our findings in humans T cells confirm our previous observations in mice in which we found RAGE expression on activated T cells [58], [59], [61]. In mice, however, we identified RAGE expression on the surfaces of diabetogenic T cells in NOD mice, but in humans we find that RAGE expression was exclusively intracellular. The receptor/ligand interactions in T cells are not clear since RAGE ligands, including S100b, advanced glycation endproducts, HMGB1 and others, are found extracellularly. One possibility is that RAGE may serve as an intracellular scavenger receptor under conditions of cellular stress and may modulate the activation and differentiation of T cells that have been previously activated or exhibit intracellular stress. For example, active secretion of HMGB1 requires the shuttling of the protein from the nucleus into the cytosol [62], [63]. There are several forms of post-translational modifications that result in the accumulation of HMGB1 in the cytosol and the protein is released via a nonclassical secretory pathway that involves specialized vesicles of the endolysosomal compartment. Therefore, it is likely that under conditions of cellular stress, HMGB1, a ligand for RAGE may be available in the cytosol. It is also possible that RAGE ligands may be available in the endosomes complexed with ligands for other receptors that are engulfed in the endosomes. This mechanism has been proposed in APCs as a means of activating endosomal TLR9 by DNA bound to the RAGE ligand HMGB1 [54].

It was unexpected to find a decrease in the level of RAGE expression following activation of T cell from patients with diabetes. The relationship between increased levels of RAGE ligands and increased RAGE expression in activated T cells suggests a “feed forward” mechanism that may explain the common finding of increased RAGE expression on T cells from patients with T1 and T2D. A limitation of our studies is that since RAGE undergoes a variety of post-translational modifications, it is possible that an isoform of the molecule is expressed by activated T cells that is not identified by the antibody we used for detection. In tissues, the full length form of RAGE is found most frequently, but RAGE undergoes a variety of splice events resulting, most commonly, in the production of a secreted form of RAGE (sRAGE) from a frameshift at the C terminus which removes transmembrane and cytoplasmic domains [19], [21], [64]. In pathologic states the level of expression of the splice variant may change which results in increased levels of sRAGE in plasma [63], [65], [66]. Nonetheless, our studies of unactivated cells indicate that there are clear differences in the patterns of RAGE expression on T cells from healthy control subjects and patients with diabetes.

RAGE appears to modulate the phenotype of CD4+ T cells. In mice, we found reduced expression of IFNγ by RAGE deficient cells and our studies with human cells show increased expression of IL-17 and CD107a in RAGE+ T cells. Signaling through RAGE has been shown to involve interactions between the FH1 domain of mammalian Diaphanous-1 that interacts with the cytoplasmic tail of RAGE and induces several intermediaries including NF-κB, MAPKs, PI3K/Akt, Rho GTPases, Jak/STAT, and Src family kinases [47], [49], [67]. Modulation of TCR signaling via these intermediates may affect the phenotype of activated cells. This observation may help to explain the observations of others concerning increased IL-17 production in responses to antigen in patients with T1DM. Nakamura et al found that circulating AGEs and sRAGE are independent determinants of serum monocyte chemoattractant protein-1 (MCP-1) levels in patients with type 2 diabetes suggesting a direct relationship between immune cell activation and AGE levels [68]. However, since RAGE expression on T cells is affected by both the presence of ligands as well as TCR stimulation, our findings suggest a more complex control of RAGE expression than simply the availability of ligands. In addition, the increased proportion of RAGE+ CD4+ T cells in cultures with peptide presented by Class I MHC molecules raises the possibility that RAGE may be induced on neighboring T cells possibly by cell∶cell contact or by soluble factors that have not been identified.

Although RAGE expression is associated with hyperglycemia rather than the autoimmune process that causes T1D, our findings may have relevance for understanding the more rapid tempo of the disease that is seen after the development of hyperglycemia. Beta cell destruction in T1DM is believed to progress in a linear fashion from the first appearance of autoantibodies until complete elimination of β cells, the tempo of the disease increases once hyperglycemia develops but studies have shown a 10 fold greater loss of insulin secretion after development of hyperglycemia compared to before diagnosis and analysis of changes in C-peptide secretion in the DCCT suggested that tight glycemic control was associated with reduced decline in β cell function [69], [70].

In summary, we have described, for the first time, the expression of RAGE in human T cells, and have explored factors that control its expression, and the relationship between RAGE expression and T cell function. The role of RAGE in modulating the function of T cells from patients with diabetes or its role in normal T cell function warrants further investigation and suggests a new relationship whereby environmental factors may modulate adaptive immune responses.

## Materials and Methods

### Cells and cell lines/Ethics statement

Peripheral blood mononuclear cells (PBMC) were obtained from blood donors (from the New York Blood Center), healthy control subjects, and patients with T1D or T2D and healthy control subjects. The patient groups participated in studies of cellular immune responses in patients and control subjects (IRB approval # 0608001773). Hemoglobin A1c levels were measured at the time of blood draw by Northwest Research Laboratory. HEK293 and Jurkat cells were obtained from ATCC. PBMC and cell lines were frozen at the time of acquisition and thawed prior to use.

### Western blot studies

PBMC from healthy blood donors were activated with OKT3 and separated into CD4+ and CD8+ T cells with Dynal beads (Invitrogen). The cells were lysed off the beads in buffer containing 10 mMTris, ph7.5, 5 mM EDTA, 150 mM NaCl and 1% Triton X-100 for 30 min on ice. The lysates were separated on a 12% NuPage pre-cast gel (Invitrogen) run with molecular weight markers (SeeBlue2, Invitrogen) and transferred to nitrocellulose. Resulting membranes were blocked with 5% milk in PBS and incubated with anti-human Rage mouse monoclonal antibody (Abcam) overnight at 4°C. Bands were detected using HRP-Goat anti Mouse secondary antibody (Biorad) followed by SuperSignal West Pico Chemiluminescent Substrate kit from Thermo Scientific. After which, the membrane was exposed to film and developed.

### Flow cytometry

PBMC were stained with mAbs to surface molecules: CD4, CD8, CCR7, CD45RA, and CD11c (all from BD/Pharmingen) and FITC labeled anti-human RAGE (Abcam). In certain experiments, the cells were fixed and permeabilized with Cyto-fix (BD/Pharmingen) prior to staining.

Cytokine and CD107a expression was also studied in patients with T1D after activation of the cells from patients with T1D with PMA (50 ng/ml) and ionomycin (500 ng/ml) for 6 hrs in the presence of Golgi-stop. The cells were stained for cell surface markers and then fixed and permeabilized with Cyto-perm. They were then washed and stained with antibodies to IL-2, IFNγ, IL-17, TNF, IL-17A, and IL-5 (BD/Pharmingen). For staining for CD107a, the mAb was added to cells during the culture with PMA/ionomycin and then added after washing, fixation, and permeabilization. The proportion of positive cells among RAGE+ or RAGE− cells was determined by gating on CD4+ and CD8+ lymphocytes and then determining the proportion of cytokine+ cells among a RAGE+ and RAGE− gate.

### Cell cultures

Peripheral blood cells were also cultured in AIM V media with 5% FCS with EBV peptide (GLCTLVAML) with or without S100b (10 µg/ml, Sigma, St Louis, MO). After 7 days in culture, the cells were stained with antibodies to CD4, CD8, as well as with Class I MHC tetramers loaded with the EBV peptide. These cells were also fixed and permeabilized and intracellular RAGE staining was analyzed. The cells were analyzed by flow cytometry and the proportion of tetramer+RAGE+CD8+ T cells was calculated [71].

### Transfection of RAGE into cell lines

RAGE cDNA was purchased from Openbiosystems and was PCR amplified and cloned via BP Gateway recombination (Invitrogen) into the pDONR221 vector. The cDNA was then transferred via an LR Gateway recombination reaction into a modified pcDNA-DEST40 expression vector so that the C- terminus of RAGE protein is fused in frame with the N-terminus of mKate2 fluorescent protein or GFP (Evrogen, Moscow, Russia). As control vectors, the original mKate2-expressing plasmid or with a plasmid expressing GFP were used. The transfected cells were co-stained with RHOB- N-RFP (OriGene,Rockville, MD) to identify early endosomes respectively. Pictures were taken on an inverted Olympus fluorescence microscope equipped with a standard red, green, blue set of filters, using either the 10× or 20× objective. Jurkat cells were studied at 24 or 48 hrs, under the inverted fluorescent microscope after electroporation with the RAGE-GFP plasmid using a BioRad Xcell Gene Pulser.

### Statistical analysis

Multiple groups were compared by ANOVA. Comparisons of cytokine protein expression among RAGE+ and RAGE− T cells was done using a paired Student's t-test after ln transformation of the data. For IL-17A, the percentage of positive cells were compared after log transformation. The data are presented as mean±SEM. All analyses were performed using GraphPad Prism 5. Two sided tests were performed and a p<0.05 was considered statistically significant.
